# Night Work, Rotating Shift Work, and the Risk of Cancer in Japanese Men and Women: The JACC Study

**DOI:** 10.2188/jea.JE20200208

**Published:** 2021-12-05

**Authors:** Ahmed Arafa, Ehab S Eshak, Hiroyasu Iso, Isao Muraki, Akiko Tamakoshi

**Affiliations:** 1 Public Health, Department of Social Medicine, Osaka University Graduate School of Medicine, Osaka, Japan; 2 Department of Public Health, Faculty of Medicine, Beni-Suef University, Beni-Suef, Egypt; 3 Department of Public Health, Faculty of Medicine, Minia University, El-Minia, Egypt; 4 Department of Public Health, Graduate School of Medicine, Hokkaido University, Sapporo, Japan

**Keywords:** night work, shift work, esophageal cancer, liver cancer, prostate cancer, cohort study

## Abstract

**Background:**

Limited epidemiological evidence has suggested a positive relationship between night shift work and the risk of cancer. Herein, we investigated the prospective association between different forms of work schedule and the risk of numerous cancers and all-cause cancer among Japanese men and women.

**Methods:**

This cohort study included 45,390 working men and women aged 40–79 years and registered in the Japan Collaborative Cohort Study (JACC Study). The Cox proportional hazards models were used to calculate the hazard ratios (HRs) and their 95% confidence intervals (CIs) for incident cancer among those who reported engagement in night work and rotating shift work for their longest occupations compared with day work.

**Results:**

Within a median follow-up duration of 14.2 years, 2,283 (9.4%) men and 1,309 (4.5%) women developed cancer. Among men, rotating shift work was significantly associated with increased risk of esophageal cancer (HR 2.47; 95% CI, 1.42–4.31) and decreased risk of liver cancer (HR 0.54; 95% CI, 0.30–0.98). Also, rotating shift work tended to be associated with the increased risk of prostate cancer (HR 1.42; 95% CI, 0.95–2.12). Night work and rotating shift work were not related to the risk of all-cause cancer in either sex.

**Conclusion:**

Rotating shift work might contribute to the increased risk of esophageal cancer and prostate cancer and the decreased risk of liver cancer among Japanese men.

## INTRODUCTION

Recently, a working group from the International Agency for Research on Cancer classified night shift work as “*probably carcinogenic to humans*” based on sufficient experimental evidence in animals but limited epidemiological evidence in humans.^1^ Since the prevalence of night shift work in Japan has been increasing,^2^ it is important to investigate the impact of night shift work on cancer risk.

Biologically, night shift work is associated with circadian rhythm disruption that suppresses melatonin; a neurohormone known for its role in hindering cancer initiation and progression.^3^^,^^4^ This disruption can also damp the expression of several circadian genes, such as *Bmal1*, *Cry1/2*, and *Dec1/2*. These genes regulate fundamental cell functions, including cell proliferation, apoptosis, metabolism, and other endocrine functions, and the dysregulation of these functions can enhance carcinogenesis.^5^ Further, night and shift workers were consistently reported to have vitamin D deficiency,^6^ and vitamin D was shown, in molecular and epidemiological studies, to play a protective role against the development of several histological types of cancer involving breast, prostate, colorectal, esophageal, and stomach cancers.^7^

While the relationship between night shift work and the risk of prostate cancer^8^^–^^12^ and breast cancer^13^^–^^20^ has been frequently assessed, researchers have failed to reach conclusive findings, while the risk of other cancers, such as lung cancer^21^^,^^22^ and colorectal cancer,^23^^,^^24^ has been scarcely investigated. For example, the Heinz Nixdorf Recall Study included men from a highly industrialized area in Germany and showed that shift work was associated with an increased risk of prostate cancer,^12^ whereas rotating shift work in the Older Finnish Twin Cohort was not associated with prostate cancer risk.^10^ Using the data of the United Kingdom Generation Study, night shift work had no relation to the risk of breast cancer in women.^20^ In contrast, the Swedish Work, Lipids, and Fibrinogen occupational cohort showed an increased risk of breast cancer in the night shift working women.^14^ In the Nurses’ Health Study I and II, no association between rotating night work and colorectal cancer risk among women was detected.^24^ A case-cohort study nested within the Shanghai Textile Industry Bureau did not reveal any increased risk of lung cancer among rotating shift workers.^22^

Three studies from the Japan Collaborative Cohort Study (JACC Study) have examined the association of shift work with incident prostate cancer^8^ and mortality from biliary tract and pancreatic cancers.^25^^,^^26^ These studies included men only and were based on short follow-up periods; nevertheless, they examined the mortality outcome as a proxy of incidence. However, data is lacking about whether night or rotating shift work can be involved in the development of esophageal, stomach, liver, and urothelial cancers. Determining these associations can help in defining a modifiable risk factor for cancer to apply risk prevention strategies and detect cohorts at risk who could be a target of screening programs. We, therefore, used the data of the JACC Study to investigate the association between different forms of work schedule and the risk of cancer incidence among Japanese men and women aged 40–79 years.

## METHODS

### Study population and baseline questionnaire

**Figure 1.**
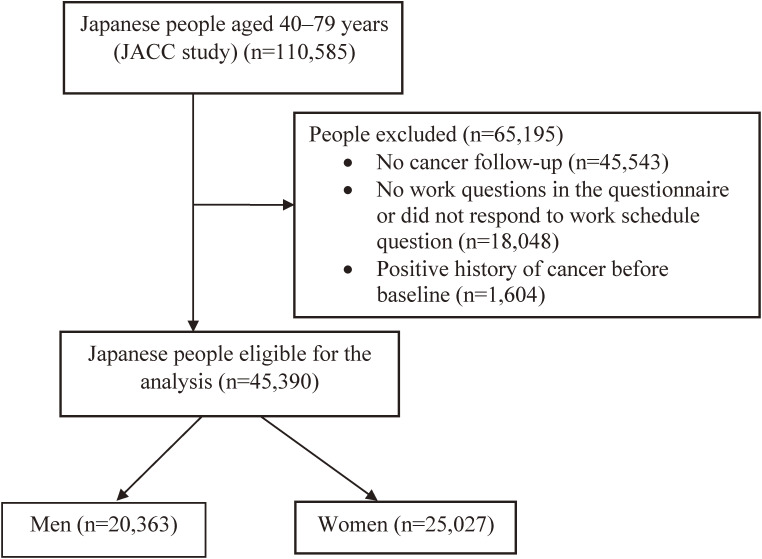
Flow chart of the study population eligibility criteria

The JACC protocol and baseline questionnaire were described elsewhere.^27^^,^^28^ Briefly, the JACC baseline data was carried out from 1988 through 1990 in 45 areas in Japan where 110,585 people (46,395 men and 64,190 women) aged 40–79 years were included. Yet, the sample of the current research was limited to 65,042 participants from 24 areas out of a total of 45 areas of the JACC study where the data of cancer incidence were available.^29^ Herein, we excluded people with a positive history of cancer before the baseline and those who did not report on the exposure status. Eventually, we confined the analysis to 45,390 participants (20,363 men and 25,027 women) (Figure 1). The JACC baseline self-administered questionnaire included data about education, employment status, physical activity, intakes of common foods and beverages, smoking and alcohol drinking habits, and past medical and obstetric histories.^29^ The sociodemographic and lifestyle characteristics of the investigated cohort in the 24 areas were similar to those of the baseline participants: body mass index (BMI) ≥25 kg/m^2^ (19.5% vs 19.6%), ever smoking (34.5% vs 35.4%), the average amount of alcohol intake/day (27.0 vs 28.8 g/day), practicing leisure sport ≥3 hours/week (10.2% vs 9.6%), and being employed (25.7% vs 23.6%).

### Ethical consideration

Informed consent was obtained before participation. The research ethics committees of Nagoya University School of Medicine and Osaka University approved the protocol of the JACC study.

### Exposure

Data on work schedule was collected using the question in the JACC Study baseline self-administered questionnaire: *“Which form of work schedule have you engaged in for your longest occupation?”*. The available responses were as follows: *“day work, night work, or alternate night and day work”*. The last choice was referred to in this article as rotating shift work.

### Covariates

Using the same questionnaire, we collected data about participants’ age, sex, weight, height, educational years, employment status, perceived stress, smoking habits and intensity, alcohol intake per day, leisure physical activities per week, walking time per day, family history of cancer, and daily intakes of several foods. Data from women on their reproductive and obstetric histories were also gathered and included.

### Outcomes

Data on cancer incidence among men and women were collected simultaneously using population-based cancer registries or by reviewing hospital records and death certificates. Participants were followed up for cancer incidence or death until the end of 2009 in four areas, 2008 in two areas, 2006 in two areas, 2003 in one area, 2002 in eight areas, 2000 in one area, 1999 in one area, 1997 in four areas, and 1994 in one area. The incidence data were coded according to the 10th revision of the International Statistical Classification of Diseases and Related Health Problems. We used the first diagnosis for incidence to include the incident cases of the following cancers: All-cause cancer (C00–C99), lung cancer (C33), esophageal cancer (C15), stomach cancer (C16), liver cancer (C22), pancreatic cancer (C25), biliary tract cancer including gallbladder and extrahepatic bile duct cancers (C23 and C24), colorectal cancer (C18, C19, and C20), urothelial cancer including renal pelvis, ureter, and urinary bladder cancers (C65, C66, and C67), breast cancer (C50), and prostate cancer (C61). Among 3,592 workers who developed cancer in this study, 30 (0.8%) cases were diagnosed by death certificate only, while the remaining 99.2% had histological verification of the diagnosed incident cancer. Because of the small number of incident cases, we could not include the following cancers in women: esophageal, biliary tract, and urothelial cancers.

### Statistical analyses

The differences in the age-adjusted mean values and proportions of personal characteristics and common risk factors for cancer among men and women with different work schedules (day, night, and rotating shift) were calculated using the linear and logistic regression tests. The Cox proportional hazards models were used to compute the sex-specific hazard ratios (HRs) and corresponding 95% confidence intervals (CIs) of the incidence of all-cause cancer and several cancers for night work and rotating shift work compared with day work. Person-years of follow-up were calculated from the date of filling out the JACC Study baseline questionnaire to the incidence of cancer, death, moving out, or end of the study, whichever came first. The HRs were adjusted for the following variables: age in years; BMI, calculated as weight in kilograms/square of height in meters (<25 and ≥25 kg/m^2^); educational level (<18 and ≥18 years); current employment status (employed, part-time, self-employed, housemaker, unemployed, and others); perceived stress (no, mild, moderate, and severe stress); smoking habits (never smokers, ex-smoker of <20 cigarettes/day, ex-smoker of ≥20 cigarettes/day, current smoker of <20 cigarettes/day and current smoker of ≥20 cigarettes/day); alcohol intake (never, ex, and current); leisure physical activity (never, 1–2, 3–4, and ≥5 hours/week); walking (never, <30, 30–60, and >60 minutes/day); family history of cancer (yes and no); and quartiles of daily intakes of protein, fat, and total energy. The regression models among women were further adjusted for the age at menarche (<15 and ≥15 years), age at first birth (<25 and ≥25 years), and baseline menopausal status (premenopausal and postmenopausal). The possibility of interaction with age category (40–59 vs 60–79 years), BMI category (<25 vs ≥25 kg/m^2^), and smoking behavior (ever vs never smokers) was examined whenever a significant association was detected. SAS version 9.4 software (SAS Institute Inc, Cary, NC, USA) was used for statistical analyses.

## RESULTS

Of the 20,363 included men, 16,267 (79.9%) were day workers, 1,535 (7.5%) night workers, and 2,561 (12.6%) rotating shift workers while among the 25,027 included women, 22,317 (89.2%) were day workers, 875 (3.5%) night workers, and 1,835 (7.3%) rotating shift workers. Within a median follow-up period of 14.2 years, 2,283 (9.4%) men and 1,309 (4.5%) women developed cancer and the incidence of cancer by work schedule was distributed as follows: among men, day workers (9.4%), night workers (9.7%), and rotating shift workers (8.8%); and among women, day workers (4.5%), night workers (5.8%), and rotating shift workers (4.7%) (data not shown in table).

Compared with day working men and women, night working men and women were older and less educated. They also reported more current smoking and alcohol intake but less perceived stress and family history of cancer. Rotating shift working men and women reported more current smoking and alcohol intake than the day working men and women (Table 1).

**Table 1.** Age-adjusted sociodemographic characteristics of Japanese working men and women distributed by their work schedule (JACC Study)

	**Day work**	**Night work**	**Rotating shift work**	***P-value***
**Men**				
**Study population**	16,267	1,535	2,561	—
**Age** ^*^	57.1 (10.1)	58.1 (10.4)	55.6 (10.2)	<0.001
**Body mass index** ^*^	22.6 (2.8)	22.8 (2.9)	22.9 (2.8)	<0.001
**High education%**	20.1	9.5	17.1	<0.001
**Employed%**	43.3	43.6	43.9	0.002
**Perceived high stress%**	25.2	21.2	27.9	0.494
**Current smoking%**	52.0	54.0	55.0	<0.001
**Alcohol intake g/day** ^*^	32.2 (21.5)	35.6 (22.5)	34.8 (22.6)	<0.001
**Leisure sport ≥3 hr/week%**	14.3	12.4	15.1	0.201
**Walking ≥60 min/day%**	11.5	10.9	11.9	0.420
**Protein intake g/day** ^*^	53.2 (15.4)	56.9 (15.8)	51.8 (15.7)	0.097
**Fat intake g/day** ^*^	31.9 (10.7)	33.3 (10.8)	31.1 (11.0)	0.126
**Energy intake Kcal/day** ^*^	1,710.5 (472.1)	1,812.5 (482.0)	1,681.4 (474.7)	0.098
**Family history of cancer%**	17.8	14.5	15.9	0.002
**Women**				
**Study population**	22,317	875	1,835	—
**Age** ^*^	56.4 (9.9)	60.2 (10.3)	58.6 (10.5)	<0.001
**Body mass index** ^*^	22.8 (3.0)	22.9 (3.2)	23.1 (3.2)	<0.001
**High education%**	10.4	7.5	11.3	0.191
**Employed%**	19.3	9.1	12.8	<0.001
**Perceived high stress%**	22.2	19.6	25.2	0.059
**Current smoking%**	5.2	11.0	9.6	<0.001
**Alcohol intake g/day** ^*^	9.2 (11.8)	21.0 (26.6)	12.2 (16.8)	<0.001
**Leisure sport ≥3 hr/week%**	9.4	10.7	12.5	0.004
**Walking ≥60 min/day%**	9.4	9.3	8.7	0.269
**Protein intake g/day** ^*^	51.8 (14.4)	51.7 (15.4)	52.1 (15.8)	0.291
**Fat intake g/day** ^*^	32.4 (10.7)	31.8 (10.9)	32.7 (11.6)	0.208
**Energy intake Kcal/day** ^*^	1,413.8 (354.5)	1,405.1 (377.3)	1,400.0 (360.4)	0.310
**Family history of cancer%**	19.1	16.8	17.0	0.034
**Age at menarche** ^*^	14.8 (1.8)	15.3 (1.8)	14.9 (1.8)	0.012
**Age at 1^st^ birth** ^*^	25.0 (3.2)	25.7 (3.3)	25.3 (3.4)	<0.001
**Menopause%**	64.8	67.0	67.4	<0.001

^*^Mean (standard deviation) for all such variables.

In the multivariable-adjusted model, rotating shift work in men was significantly associated with the increased risk of esophageal cancer (HR 2.47; 95% CI, 1.42–4.31) but with the reduced risk of liver cancer (HR 0.54; 95% CI, 0.30–0.98). These associations showed no interaction with age, BMI, and smoking behavior (*P*-interaction >0.10). Rotating shift work tended to be associated with the increased risk of prostate cancer (HR 1.42; 95% CI, 0.95–2.12) (Table 2). No significant associations were detected between night work or rotating shift work and the risk of all-cause cancer or any other cancers in men and women (Table 2 and Table 3).

**Table 2.** Association of night work and rotating shift work with cancer incidence among Japanese men (JACC Study)

	**Day work**	**Night work**	**Rotating shift work**
**Person-years**	212,092	21,771	33,407
**Study population**	16,267	1,535	2,561
**All-cause cancer incidence**			
**Incident cases**	1,999	212	293
**Model I**	1	0.93 (0.80–1.07)	1.02 (0.90–1.16)
**Model II**	1	0.92 (0.80–1.07)	1.03 (0.91–1.16)
**Lung cancer incidence**			
**Incident cases**	281	23	30
**Model I**	1	0.69 (0.45–1.06)	0.75 (0.52–1.10)
**Model II**	1	0.71 (0.46–1.09)	0.77 (0.53–1.13)
**Esophageal cancer incidence**			
**Incident cases**	50	8	17
**Model I**	1	1.46 (0.69–3.08)	2.30 (1.32–3.99)
**Model II**	1	1.63 (0.77–3.48)	2.47 (1.42–4.31)
**Stomach cancer incidence**			
**Incident cases**	551	64	83
**Model I**	1	1.03 (0.79–1.33)	1.05 (0.83–1.32)
**Model II**	1	0.97 (0.75–1.27)	1.05 (0.83–1.32)
**Liver cancer incidence**			
**Incident cases**	147	11	12
**Model I**	1	0.67 (0.37–1.25)	0.57 (0.32–1.03)
**Model II**	1	0.63 (0.34–1.16)	0.54 (0.30–0.98)
**Pancreatic cancer incidence**			
**Incident cases**	50	5	9
**Model I**	1	0.84 (0.33–2.10)	1.32 (0.65–2.68)
**Model II**	1	0.92 (0.36–2.33)	1.33 (0.65–2.71)
**Biliary tract cancer incidence**			
**Incident cases**	46	6	10
**Model I**	1	1.10 (0.47–2.58)	1.58 (0.80–3.13)
**Model II**	1	0.96 (0.40–2.31)	1.51 (0.76–3.00)
**Colorectal cancer incidence**			
**Incident cases**	235	16	35
**Model I**	1	0.62 (0.37–1.03)	1.01 (0.71–1.44)
**Model II**	1	0.64 (0.38–1.06)	1.04 (0.73–1.49)
**Urothelial cancer incidence**			
**Incident cases**	86	12	9
**Model I**	1	1.21 (0.66–2.21)	0.74 (0.37–1.47)
**Model II**	1	1.35 (0.73–2.49)	0.75 (0.38–1.50)
**Prostate cancer incidence**			
**Incident cases**	150	27	29
**Model I**	1	1.42 (0.94–2.14)	1.42 (0.95–2.12)
**Model II**	1	1.36 (0.89–2.07)	1.42 (0.95–2.12)

Model I: Adjusted for age.

Model II: Further adjusted for body mass index, education, employment status, perceived stress, smoking behavior, alcohol, leisure physical activity, walking, family history of cancer, and intakes of protein, fat, and total energy.

**Table 3.** Association of night work and rotating shift work with cancer incidence among Japanese women (JACC Study)

	**Day work**	**Night work**	**Rotating shift work**
**Person-years**	290,746	12,452	23,915
**Study population**	22,317	875	1,835
**All-cause cancer incidence**			
**Incident cases**	1,296	72	113
**Model I**	1	1.07 (0.84–1.36)	0.95 (0.79–1.16)
**Model II**	1	1.05 (0.83–1.33)	0.93 (0.77–1.13)
**Lung cancer incidence**			
**Incident cases**	99	4	4
**Model I**	1	0.72 (0.26–1.96)	0.43 (0.16–1.17)
**Model II**	1	0.70 (0.25–1.90)	0.43 (0.16–1.17)
**Stomach cancer incidence**			
**Incident cases**	273	20	27
**Model I**	1	1.35 (0.85–2.13)	1.05 (0.71–1.56)
**Model II**	1	1.34 (0.85–2.13)	1.05 (0.70–1.56)
**Liver cancer incidence**			
**Incident cases**	69	3	7
**Model I**	1	0.73 (0.23–2.33)	1.04 (0.48–2.27)
**Model II**	1	0.75 (0.24–2.42)	1.00 (0.46–2.18)
**Pancreatic cancer incidence**			
**Incident cases**	58	4	6
**Model I**	1	1.14 (0.41–3.15)	1.05 (0.45–2.44)
**Model II**	1	1.05 (0.38–2.92)	1.02 (0.44–2.37)
**Colorectal cancer incidence**			
**Incident cases**	176	9	19
**Model I**	1	0.91 (0.46–1.78)	1.12 (0.70–1.81)
**Model II**	1	0.88 (0.45–1.73)	1.08 (0.67–1.74)
**Breast cancer incidence**			
**Incident cases**	151	7	13
**Model I**	1	1.18 (0.55–2.52)	1.08 (0.61–1.90)
**Model II**	1	1.18 (0.55–2.54)	1.02 (0.57–1.80)

Model I: Adjusted for age.

Model II: Further adjusted for body mass index, education, employment status, perceived stress, smoking behavior, alcohol, leisure physical activity, walking, family history of cancer, intakes of protein, fat, and total energy, age at menarche, age at first birth, and menopausal status.

## DISCUSSION

This prospective cohort study indicated that rotating shift working men, compared with day working men, were more likely to develop esophageal cancer (HR 2.47) but less likely to develop liver cancer (HR 0.54). Also, rotating shift work tended to be associated with an increased risk of prostate cancer (HR 1.42). On the other hand, night work and rotating shift work were not associated with the risk of all-cause cancer in men and women or any type of cancer in women.

Previous research focused on investigating the impact of night shift work on the development of prostate, breast, lung, and colorectal cancers. Our results came in line with previous cohort studies that showed an increased risk of prostate cancer,^8^^,^^9^^,^^12^ but no excess risk of breast,^13^^,^^15^^–^^18^^,^^20^ lung,^22^ or colorectal cancers^23^^,^^24^ among shift workers. However, our results disagreed with other studies.^10^^,^^11^^,^^14^^,^^21^ It should be noted that the previous studies were conducted on populations with different sociodemographic characteristics and used dissimilar definitions and data collecting methods for night shift work that may explain their inconsistent findings.

Besides, previous studies used the data of the JACC study to assess possible relationships between shift work and the risk of cancer incidence and mortality in men.^8^^,^^28^^,^^29^ Kubo et al, similar to our findings, detected a significant risk of prostate cancer among rotating shift workers compared with day workers (HR 3.0; 95% CI, 1.2–7.7). However, their study was limited by the relatively short period of follow-up (8 years vs 14.2 years in this study), and the small number of prostate cancer cases (31 cases vs 206 cases in this study).^8^ In two other studies using the mortality data of the JACC study as a proxy of incidence, Lin et al, similar to our findings on cancer incidence, showed no significant associations between rotating shift work and the risk of mortality from biliary tract and pancreatic cancers in Japanese men.^25^^,^^26^

Several underlying mechanisms can explain the relationship between rotating shift work and the risk of cancer. One of these mechanisms is that the exposure to light at night and the disruption of circadian rhythm reduce melatonin production.^3^^–^^5^ Melatonin has oncostatic property via antioxidant activity, apoptosis stimulation, free radical scavenging, and angiogenesis inhibition.^3^^,^^4^ It could be even used as adjuvant therapy for prostate, breast, stomach, and colorectal cancers.^30^ Moreover, night shift workers are less exposed to sunlight and suffer vitamin D deficiency.^6^ Vitamin D regulates several cell behaviors including proliferation, differentiation, apoptosis, and autophagy, and their dysregulation can induce carcinogenesis.^7^ Previous ecological and epidemiological studies showed that sunlight exposure and vitamin D levels were inversely associated with the incidence of several cancers such as colorectal, pancreatic, esophageal, and oropharyngeal cancers, in addition to all-cause cancer incidence and mortality.^31^^,^^32^ Also, consuming snacks after midnight at irregular times; an eating habit characterizing night shift workers, was shown to be associated with increased levels of 8-isoprostane,^33^ a marker of oxidative stress that contributes to the development of numerous cancers.^34^

In terms of prostate cancer, melatonin showed antiproliferative action on human prostate cancer cells.^35^ Independent of melatonin, increased levels of androgens, that can play a role in the development of prostate cancer, were observed among night shift workers compared with day workers.^36^ Additionally, strong evidence of a positive association between shift work and elevated prostate-specific antigen levels was documented in the National Health and Nutrition Examination Survey (NHANES) study indicating that shift work would likely increase the risk of prostate cancer.^37^

To our knowledge, this study is the first to detect a significant association between rotating shift work and the risk of esophageal cancer among men. Although rotating shift workers in the JACC study reported more established risk factors for esophageal cancer such as high BMI, current smoking, and alcohol intake compared with fixed day workers,^38^ adjusting for these factors did not reduce the risk. In a previous case-control study from Canada, limited evidence of an association between night work and esophageal cancer incidence in men was suggested (Odds ratio 1.51; 95% CI, 0.80–2.84).^39^ One of the main shortcomings of the Canadian study was that they did not differentiate between fixed night work and rotating shift work. In our study, the risk of developing esophageal cancer among night workers (HR 1.63; 95% CI, 0.77–3.48) was significantly lower than that of rotating shift workers (HR 2.47; 95% CI, 1.42–4.31). Also, the study used the electoral lists to recruit controls who could have carried different characteristics compared with the patients.^39^

Numerous mechanisms can vindicate the increased risk of esophageal cancer among rotating shift workers. For example, certain circadian genes such as *Dec1/2* are involved in the regulation of the immunological system of the gastrointestinal tract and the restoration of the esophageal mucosal barrier.^40^ Besides, melatonin is considered an esophagoprotector, acting through preventing esophageal injury, improving esophageal blood flow, and activating the sensory nerves.^41^ Thus, circadian rhythm disruption and melatonin deficiency can contribute to the development of gastroesophageal reflux disease^41^^,^^42^; a disease characterized by insufficient sphincter function in the lower esophagus and considered a major risk factor for esophageal cancer.^43^ Moreover, vitamin D deficiency, which characterizes night shift workers, was shown to be associated with the risk of esophageal cancer since vitamin D can mitigate the inflammatory and genotoxic effects of COX-2 and reactive oxygen species that can initiate esophageal cancer.^44^^,^^45^ Interestingly, one study showed that shift workers experienced frequent apneic events during morning sleep after the night shift.^46^ Japanese night shift workers commonly use alcohol as a sleep aid because alcohol intake can initially be associated with improved sleep onset,^47^ but this effect diminishes shortly with the continued use and may even lead to greater sleep disturbances.^48^ Intermittent hypoxemia enhanced cancer progression in animal models with sleep apnea,^49^ and people with regular snoring showed an increased risk of esophageal cancer by 156% after adjusting for BMI, smoking, alcohol intake, and dietary habits.^50^ Taken together, the positive association between rotating night shift and the increased risk of esophageal cancer should not be unexpected.

Surprisingly, we could observe a modestly negative association between rotating shift work and the risk of liver cancer among men but not among women. This was an unpredicted finding because the protective effects of melatonin against several factor-induced liver injuries, fibrosis, cirrhosis, and cancer have been extensively described,^51^ and night shift work was related to liver enzyme abnormalities.^52^ However, since chronic viral hepatitis is the major risk factor for liver cancer in Japan,^53^ it could be predicted that the poor health condition of patients with chronic viral hepatitis prevents them from working night shifts. Unfortunately, we had no data on the exact jobs of the included participants; however, we controlled for the baseline employment status as a proxy of the job and the associations did not change materially. Still, this suggestion cannot explain the sex-specific differences regarding this relationship, thus, more research is needed to confirm this finding and unveil possible mechanisms.

Although this study had several strengths such as the large cohort, the exclusion of people with a previous history of cancer, the long follow-up period, the standardized approaches of cancer diagnosis, and the control for most potential confounders, some limitations should be addressed. First, according to the baseline questionnaire, the definition of rotating shift work did not describe the frequency of shifts, working hours per shift, and working tenure, therefore, the dose-response effect could not be studied. A previous meta-analysis of cohort and case-control studies showed that an increase of night shifts by 500 nights would lead to a 13% increase in the risk of breast cancer,^54^ yet this finding was challenged by a meta-analysis of prospective cohort studies which detected no excess risk of breast cancer among workers with ≥20 years of night shift work (HR 1.01; 95% CI, 0.93–1.10) or with ≥30 years (HR 1.00; 95% CI, 0.87–1.14) when compared with day workers.^18^ Second, since shift workers are at high risk of cardiovascular events, sleep disorders, and accidents,^55^ it could be speculated that they have frequent access to health facilities and consequently they are more likely to get diagnosed with cancers. However, a previous report showed that shift workers had less access to health promotion facilities and were less adherent to cancer screening recommendations.^56^ Third, it could be argued that some workers might have changed their work schedule during the follow-up suggesting a possibility of misclassification bias. But, the work schedule in this study was assessed based on the longest occupation, not the baseline one which minimizes the possibility of bias. Fourth, data on work schedules were collected using self-report that was not validated. However, a survey conducted by the Japanese Ministry of Internal Affairs and Communications showed that fixed night workers represented 5.0% of the total employed workforce in 1997,^2^ close to the 5.3% prevalence in this study. Fifth, the limited number of cases with liver cancer may have resulted in indecisive conclusions.

In conclusion, this prospective cohort study indicated that rotating shift work might be associated with increased risk of esophageal cancer and decreased risk of liver cancer among men. It also supported the concept that rotating shift work can be associated with a higher risk of prostate cancer. However, no associations were detected between rotating shift work and the risk of all-cause cancers in either sex.
